# Evaluation of oral keratinocyte progenitor and T-lymphocite cells response during early healing after augmentation of keratinized gingiva with a 3D collagen matrix - a pilot study

**DOI:** 10.1186/s12903-016-0240-x

**Published:** 2016-07-07

**Authors:** Darian Rusu, Bogdan Calenic, Maria Greabu, Alexander Kralev, Marius Boariu, Florina Bojin, Simona Anghel, Virgil Paunescu, Octavia Vela, Horia Calniceanu, Stefan-Ioan Stratul

**Affiliations:** Department of Periodontology, Victor Babes University of Medicine and Pharmacy, Bv. Revolutiei 1989, Nr. 9, 300041 Timisoara, Romania; Department of Biochemistry, Faculty of Dental Medicine, University of Medicine and Pharmacy, Carol Davila, Blvd-ul Eroii Sanitari, No 8, Bucharest, Romania; Victor Babes National Institute of Pathology, Biochemistry-Proteomics Department, Blv. Splaiul Independenţei nr. 99 - 101, Bucharest, Romania; Department of Odontotherapy and Endodontics, Victor Babes University of Medicine and Pharmacy, Bv. Revolutiei 1989, 9, 300041 Timisoara, Romania; Department of Functional Sciences-Immunology, Victor Babes University of Medicine and Pharmacy, Pta Eftimie Murgu nr.2, 300041 Timisoara, Romania; Dental Clinic Dr.Stratul, Str.Emanoil Gojdu, nr.5, 300176 Timisoara, Romania; Department of Dentistry, Faculty of Medicine and Pharmacy, University of Oradea, Pta 1 Decembrie nr.10, Oradea, Romania

**Keywords:** Keratinocytes, T-lymphocytes, Oral mucosa, Collagen matrix

## Abstract

**Background:**

The aim of the present study is to analyze the behavior of selected populations of oral keratinocytes and T-lymphocytes, responsible for re-constructing and maintaining the oral epithelial tissue architecture, following augmentation of the keratinized oral mucosa using a 3D-collagen matrix.

**Methods:**

Different groups of oral keratinocytes were isolated from biopsies harvested from 3 patients before the surgical procedure, as well as 7 and 14 days after the augmentation procedure. T-lymphocytes were isolated from peripheral blood at same timepoints. Keratinocytes were characterized for stem and differentiation markers, such as p63, cytokeratin 10 and 14, and in vitro parameters, such as cell viability, cell size and colony-forming efficiency. T-lymphocytes were analyzed for viability and the expression of various cluster of differentiation markers. The methods included magnetic separation of cell populations, immunofluorescence, flow cytometry, and histology of oral biopsies.

**Results:**

Both at 7 and 14 days, the majority of cells that repopulate the matrix were actively proliferating/progenitor oral keratinocytes with the phenotype integrin alfa6beta4 + CD71+. These cells display in vitro characteristics similar to the progenitor cells analyzed before the matrix placement. T-lymphocytes expressed CD8 and CD69 markers, while CD25 was absent.

**Conclusion:**

The study shows that two weeks after the collagen membrane placement, the healing process appeared to be histologically complete, with no abnormal immune response induced by the matrix, however, with a higher than usual content of active proliferating cells, the majority of keratinocytes being characterized as transit amplifying cells.

## Background

A healthy and functional mucogingival complex including a band of keratinized gingiva is considered to be important to ensure the underlying osseous stability around teeth and implants [1]. Moreover, a lack of adequate keratinized mucosa around dental implants is associated with more plaque accumulation, tissue inflammation, mucosal recession as well as loss of attachment [2], therefore, soft tissue augmentation procedures in clinical practice have been the focus of increased attention recently. The rationale for performing gingival augmentation procedures around natural teeth and dental implants includes facilitating plaque control and improving patient comfort, in conjunction with restorative, prosthetic or orthodontic procedures, and the prevention of gingival recession [3]. The surgical approach most frequently used for gingival augmentation is the apically repositioned flap plus the application of an autogenous free gingival graft or subepithelial connective tissue graft harvested from the palatal mucosa [4].

Over the past decades, complete regeneration of the oral mucosa following surgery has been the subject of intense research and debate [5, 6]. In order to avoid morbidities at the donor site, allograft materials, such as acellular dermal matrix grafts and human fibroblast-derived dermal substitutes, have also been used as an alternative [7, 8]. Recently, a two-layer xenogeneic collagen matrix has been used for augmenting keratinized tissue around teeth and dental implants [1, 9, 10], while a systematic review concluded that the apically positioned flap/vestibuloplasty plus collagen matrix demonstrated less gain in keratinized mucosa, but also less patient morbidity and surgery time [11].

Prolonged healing times, limited functional and mechanical properties, immature basal lamina and poor anchoring areas still need to be addressed when discussing grafting procedures of oral mucosa [12]. Re-epithelization following trauma or surgery represents an essential process that is initiated by oral keratinocytes situated around the wound, which are activated by a variety of signal molecules and start migrating to the wound site [13]. Previous experimental models show that activation of the immune system and especially T-lymphocytes (TL) represents a requirement for the optimal healing following wounds or surgery in the oral cavity [11].

The epithelia consist of several layers of keratinocytes that have different characteristics. For detailed discussions on keratinocyte cells and their specific roles, several comprehensive reviews are available [14–20]. The general architecture of the oral epithelium includes oral keratinocyte stem cells found close to the basal membrane, and to the blood vessels of the lamina propria. These cells possess several attributes that clearly distinguish them from other oral keratinocytes: self renewal potential, ability to differentiate into other cell types from the same tissue, quiescence and a long life span. They divide asymmetrically and give rise to progenitor cells called “transit amplifying (TA) cells” that are actively proliferating cells and migrate from the lower suprabasal layers to the upper ones. Previous studies show that the proportion of progenitor cells in the mammalian epithelium varies from 1 % [21] to 15–20 % [22–24]. Specifically, in oral epithelia, the percentage of progenitor cells is usually lower than in the epidermis [25]. Our previous results demonstrate that in normal oral epithelia, TA oral keratinocytes are a subpopulation consisting of approximately 15 % of the total oral keratinocytes [26–29]. Following several rounds of cellular division, these cells differentiate to post-mitotic keratinocytes. Due to their intrinsic attributes, progenitor cells might be actively involved in regenerating the epithelial bed following reconstructive surgery or trauma [30–33].

Oral keratinocyte progenitor cells are also known to replace damaged epithelia with similar traits throughout the human body. Progenitor cells have been shown to promote epithelization in animal esophagus, regenerate human cornea in transplantation procedures [34], act as adjuvant for treating limbal stem cell deficiencies and are the elective treatment for urethroplasty procedure [35, 36]. While several studies focused on the in vitro and in vivo behavior of oral fibroblasts following gingival augmentation, data on oral keratinocytes is scarce [37, 38]. A recent in vitro study evaluated cellular functions, such as adhesion, IL-6 production and proliferation of human gingival keratinocytes cultured on a newly engineered collagen matrix (CM-10826) and the assessment of the degree of specific biocompatibility of this new device [37]. Due to previous difficulties in isolating and characterizing different oral keratinocyte sub-populations, a thorough analysis of the cellular behavior following contact with various oral soft tissue substitutes is lacking. The hypothesis of the present study is that the cytological composition of different populations of keratinocytes does not follow the clinical and histological healing of the keratinized mucosa augmented with collagen substitutes.

Our group managed to successfully separate and define several sub-groups of oral keratinocytes, based on two surface markers (integrin alfa6beta4 and CD71), demonstrating the existence of three separate populations: oral keratinocyte stem cells (OKSCs), TA cells and post-mitotic differentiation cells [45–47]. The specific aim of the present study is to analyze two cell types playing key roles in healing of oral mucosa following a routine surgical procedure consisting of placing a resorbable 3D collagen matrix used for oral soft tissue regeneration. The study will assess the behavior of different sub-groups of oral keratinocytes and of inflammatory cells, such as T-lymphocytes, in contact with the collagen matrix. The study is particularly important from a clinical point of view as, on one hand, it demonstrates disparities between the clinical and histological aspects of the augmented gingiva, and, on the other hand, the cytologic level of maturation, as assessed in vitro.

## Methods

The study was designed as a pilot study for a harvesting procedure that simultaneously allows both cell cultures and histological assessment from gingival biopsies of very reduced dimensions, in an attempt to minimize any possible disturbance of the healing process.

### Patient selection

Biopsies from three different patients were included in this study. The subjects were selected from the outpatients of the Department of Periodontology of the Victor Babes University of Medicine and Pharmacy of Timisoara, Romania. They met the following inclusion criteria: at least 2 but not more than 4 adjacent inferior teeth (up to the 2nd premolar) with less than 2mm of mucosa that required augmentation; no need of root coverage at the time of grafting. The exclusion criteria employed for each patient were: active periodontal disease, smokers, patients with history of alcohol abuse, systemic diseases, known alergies, pregnacies, and acute or chronic infections. The patients’ background information was as follows: Patient 1 (Sample 1) - 38 yo, female, biopsy site: teeth – 3.2–4.3; Patient 2 (Sample 2)–52 yo, female, biopsy site: teeth 3.3–3.5; Patient 3 (Sample 3)–56 yo, female, biopsy site: teeth 4.3–4.5.

### Clinical procedures

**Fig. 1:**
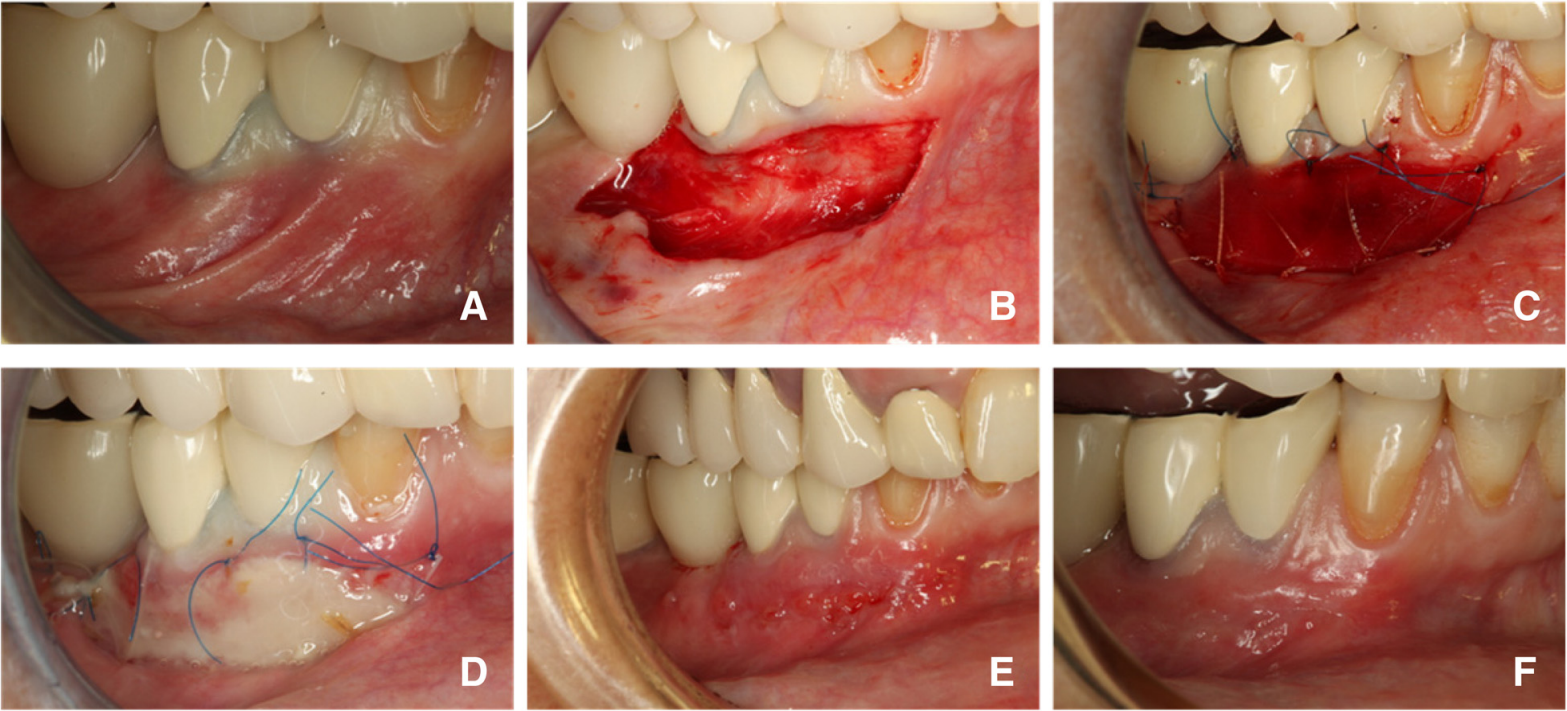
Images describing the surgical procedure: **a**) initial situation with deficit of keratinized gingiva; **b**) mucosal fenestration with apically positioned flap; **c**) the collagen matrix sutured in place; **d**) one week after the surgery; **e**) ten days after the surgery, immediately after the removal of the sutures; **f**) two weeks after the surgery

Prior to surgery, demographics, medical and dental histories were collected. An oral examination and professional cleaning were performed, clinical measurements were done, x-rays and photographs were taken, oral hygiene procedures reviewed and reinforced. Alveolar bone level and surgical position limits were obtained at baseline. The first follow-up visit occurred one week post-surgery and the second visit one week later. At each visit, biopsies were taken and sent to the cell culture laboratory for further analysis. The surgical procedure consisted of placing a fully resorbable 3D collagen matrix designed for soft tissue regeneration (Geistlich Mucograft*®,* Geistlich Pharma AG, Wolhusen, Switzerland) at the surgical site using a modification of a well-known protocol [10, 39]. Briefly, after local anesthesia, a coronal incision was made at the muco-gingival junction extending at least to the line angle of the adjacent teeth, and vertical incisions were made at both the mesial and distal aspects of the grafted sites, so that rectangular wound beds were slightly larger than the collagen matrix. A partial-thickness flap was performed, was displaced apically and was sutured with 6-0 resorbable sutures. Muscle fibers were removed to expose the periosteal bed. The collagen matrix was cut to fit the recipient site, was placed dry and was sutured in place with single non-resorbable and resorbable6-0 sutures disposed circumferentially, so that the matrix soaked with blood would stabilize the clot over the wound bed. Lips and cheek adjacent to the grafted sites were put under tension, to ensure there was no traction on the operated areas. (Figures 1 a-d). Patients were instructed to use chlorhexidine 0.12 % mouth rinse for 30 s twice daily, to avoid aggressive rinsing or brushing of the grafted area and hard foods for two weeks after the surgery. Sutures were removed after ten days. After two weeks, brushing was resumed using soft brushes and delicate movements to avoid any trauma. Normal brushing was resumed after six weeks.

### Biopsy harvesting procedure

Following a protocol described in the literature [10], biopsies of full-depth mucosa (down to the bone level) from pristine keratinized gingival areas and newly formed keratinized gingiva were harvested under local anesthesia using a 3-mm biopsy punch, prior to surgery, after 7 and after 14 days, for a different histological study (data to be published). A part of each sample was used for cell cultures in the present study, the rest was used for further detailed histological analysis.

All biopsies were performed from the central zone of the grafted area under the dental operating microscope with the aid of microsurgical instruments to avoid any disturbance of the healing process. To determine the exact region of harvesting and to avoid harvesting twice from the same site, preoperative and postoperative photographs were taken and surgical sketches were drawn. Specimens were fixed in buffered 4 % formaldehyde and sent to the histology laboratory. The fixed biopsies were oriented in a colored-coded biomimetic gel (BiopsyBoat™, Themis Pathology SRL, Bucharest, Romania), post-fixed with formal calcium, dehydrated in graded ethanols, and embedded in celloidin-parrafin. Semi-serial sectioning was performed at 5 μm and the resulting sections were stained with hematoxilin-eosin (HE).

### Immuno-magnetic isolation of oral keratinocyte progenitor cells

Cell culture protocols and cell separations were performed using a protocol described in detail by Calenic et al [40, 41]. Briefly, biopsies were rinsed with phosphate buffer saline at pH 7and subjected to enzymatic dissociation in Collagenase (Sigma, St. Louis, MO) and Dispase (Sigma, St. Louis, MO) at 4^0^C overnight. Following primary culture, the cells were separated using MACS (Magnetic Activated Cell Sorting, MACS Miltenyi Biotec, Bergisch Gladbach, Germany) and two surface markers: CD71 and integrin α6β4 (Mouse monoclonal [450–30A] antibody to Integrin alpha 6 beta 4 (Abcam) conjugated with Fluorescein isothiocyanate (FITC); goat anti-mouse IgG MicroBeads and CD71 MicroBeads). After both separation steps, three cell fractions were obtained: α6β4neg, α6β4pos CD71pos and α6β4 pos CD71neg fraction. In the present study, we analyzed α6β4pos CD71pos fraction, which, in our previous studies, demonstrated important progenitor cells attributes. The cells were further grown in Petri dishes pre-coated with human collagen IV (Sigma) (20μg/ml).

### Viability of oral keratinocyte progenitor cells

For oral keratinocyte progenitor cells, cellular viability was assayed using Trypan blue exclusion. Briefly, Trypan blue stains dead cells in blue; thus the number of dead blue cells among the total number of cells was counted. For statistical purposes the assay was performed five times.

### Colony forming efficiency of oral keratinocyte progenitor cells

In order to evaluate colony forming efficiency, 1x10^4^ cells were seeded on type IV-collagen as described above; after 14 days, the cells were fixed and stained with crystal violet. Colonies with more than 20 cells were counted with Cell Analyst (AssaySoft Inc., Fountain Valley, CA, USA). For statistical purposes the assay was performed five times.

### Immunofluorescence of oral keratinocyte progenitor cells

Immunofluorescence staining techniques followed traditional well-established protocols. Thus, following isolation, the cells were fixed using 4 % paraformaldehyde and further permeabilized with Triton X 100, followed by staining using a selection of primary antibodies, and finally by labeled secondary antibodies (as described in detail in section Antibody Library). For negative controls, the primary antibody was omitted during the immunofluorescence staining procedure. All samples were further observed under a confocal scanning laser fluorescent microscope.

### Cell size of oral keratinocyte progenitor cells

Photographs of OKSCs populations were taken using a light microscope, and the images were analyzed using Cell Analyst (AssaySoft Inc., Fountain Valley, CA, USA). The assay was repeated five times, with 20 cells being counted for each experiment.

### Antibody library

For magnetic isolation, the following antibodies were used: MicroBeads conjugated to anti-human CD71 (Miltenyi Biotec, Inc., Auburn, CA, USA); mouse monoclonal [450-30A] antibody to integrin a6b4 (Abcam, Germany) and anti-mouse IgG MicroBeads (Miltenyi Biotec Inc., Auburn, CA, USA). For immunofluorescence staining the following antibodies were used: mouse monoclonal anti-p63 (Santa Cruz Biotechnology, Inc., Santa Cruz, CA, USA); mouse monoclonal anti-cytokeratin (CK) 10 (Acris GmbH, Hertford, Germany); mouse monoclonal anti-CK14 (Sigma-Aldrich, Germany). Primary antibodies were diluted at 1: 200. Alexa Fluor—conjugated donkey anti-mouse (Invitrogen, Eugene, OR) was used as a secondary antibody. Nuclei staining was done with 4′,6-diamidino-2-phenylindole (DAPI) (Invitrogen, Eugene, OR, USA).

### T Lymphocytes isolation

Samples of 10ml of peripheral venous blood were collected on anticoagulant [Heparin 15000 IU/5mL, Biochemie GmbH, Kundl, Austria]. Separation of mononuclear cells (PBMCs—Peripheral Blood Mononuclear Cells) from peripheral blood samples was performed by centrifugation on Ficoll-Paque™ Plus (GE Healthcare Bio-Sciences AB, Uppsala, Sweden) gradient. After centrifugation, the supernatant was removed, and the cell pellet was used for in vitro analysis or frozen at -80 °C and kept for further investigation. Part of the PBMCs obtained in the previous step were cultured in order to increase the population of T-lymphocytes for 48 h in a specific culture medium (T-Cell expansion Stemline media, Sigma-Aldrich), supplemented with 10 ml L-Glutamine 200 mM/500 ml medium. After 48 h of in vitro cultivation, the cells were placed in contact with the 3D collagen matrix (1 x 10^5^ T cells/mm^3^ collagen matrix) and transferred to 24-well plates. The comparative analysis was performed between the cells in contact with the 3D collagen matrix and the control-group cells.

### T Lymphocytes viability

For the determination of induction and apoptotic execution propensity, the Annexin-V/PI method was used. In order to distinguish apoptotic cells from cells with permeabilized plasma membrane during the late non-apoptotic death, a viability marker (propidium iodide) was used, so that only apoptotic cells appeared positive for annexin V, and double-positive cells (annexin V+ iodide Propidium+) were eliminated.

### Immunophenotyping analysis of T-cells by flow-cytometry

Lymphocyte immunophenotyping analysis was carried out both for lymphocytes cultured in the main culture medium, as well as for lymphocytes which have been in contact with the collagen matrix for 5 days. Surface markers were evaluated by flow cytometry after preparing the cells for this purpose. The cells, with a 10^5^ cells/ml concentration, were washed in PBS, resuspended in PBS and incubated for 30 min in the dark with fluorochrome-conjugated monoclonal antibodies using the manufacturers recommended dilutions. After washing with a dedicated solution (Cell Wash Solution, BD Biosciences, San Jose, CA, USA), the cells were resuspended in 500 μl Cell Wash and analyzed with a FACSCalibur flowcytometer (BD Biosciences, San Jose, CA, USA). Data acquisition was performed using the program CellQuest Pro software (BD Biosciences, San Jose, CA, USA), and data analysis using the free flow cytometry data analysis Flowing Software 2.5.

### Statistical analysis

For the oral keratinocytes in vitro behaviour, results from five independent experiments are shown as means ± SD. For each independent experiment, the same number of cells was used. Statistical analysis was performed using Student’s *t*-test. Statistical significance was accepted at *p <* 0.05. For T-lymphocytes data, Student’s *t*-test was employed for all data.

## Results

The healing occurred uneventfully in all patients. At seven days after the surgery, the recipient site appeared still covered with a fibrin-like layer of fragile aspect, with several areas of flat granulation appearance (Fig. 1d). At 14 days after the surgery, the recipient site displayed a macroscopical aspect of complete healing (Fig. 1f).

### Oral keratinocyte progenitor cells

Cell count following magnetic separation demonstrated that, as opposed to normal oral mucosa, at both 7 and 14 days, most of the cells (over 90 %) were found to have the alfa6beta4 + CD71+ phenotype, which identifies the oral keratinocyte progenitor cells subgroup.

#### Cell viability

**Fig. 2:**
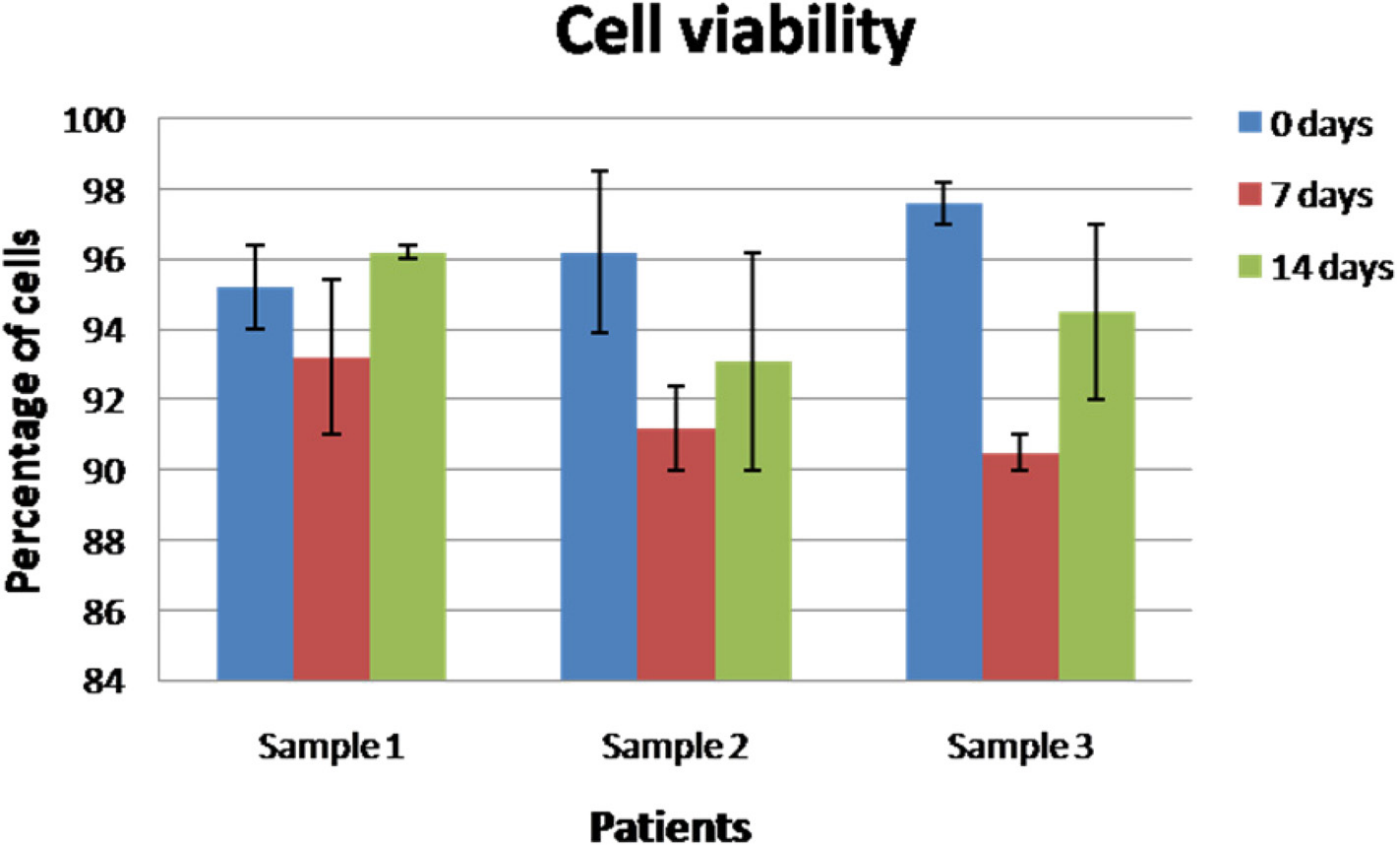
Cell viability. Graph representing the percentage of α6β4+/CD71+ cells cells at different time points: 0, 7 and 14 days. At each analyzed time point most cells were found viable in all analyzed samples, with rare necrosis (each sample identifies different individuals; *n =* 5, *p* <0.05 Student’s *t*-test)

The data represent the percentage of viable oral keratinocyte progenitor cells for various samples at different time points: 0, 7 and 14 days. At each analyzed point in time, most cells were found to be viable, as follows: Sample 1–95.2 ± 1.2 vs. 93.2 ± 2.2 vs. 96.2 ± 0.2; Sample 2–96.2 ± 2.3 vs. 91.2 ± 1.2 vs. 93.1 ± 3.1; Sample 3–97.6 ± 0.6 vs. 90.5 ± 0.5 vs. 94.5 ± 2.5 for 0, 7 and 14 days, respectively (*n =* 5, *p* <0.05 Student’s *t*-test) (Fig. 2).

#### Colony forming efficiency

**Fig. 3:**
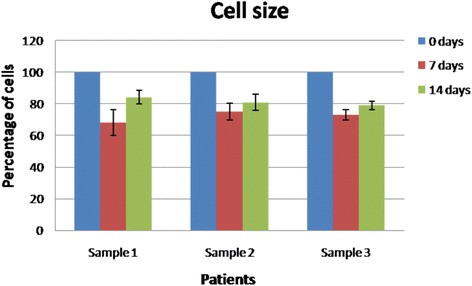
Colony forming efficiency. Graph representing the colony forming potential of α6β4+/CD71+ cells at baseline, 7 and 14 days, respectively (number of CFUs). Following magnetic separation, cells were stained with crystal violet; colonies larger than 20 cells were counted individually using an image software (each sample identifies different individuals; *n =* 5, *p* <0.05 Student’s *t*-test)

Colony forming efficiency was analyzed at 0, 7 and 14 days for each sample and the data represent the number of CFUs, as follows: Sample 1–194 ± 14 vs. 144 ± 4 vs. 178 ± 8; Sample 2–201 ± 15 vs. 125 ± 5 vs. 189 ± 9; Sample 3–183 ± 13 vs. 123 ± 12 vs. 180 ± 10 for each time point respectively (*n =* 5, *p*<0.05 Student’s *t*-test) (Fig. 3).

#### Cell size

**Fig. 4:**
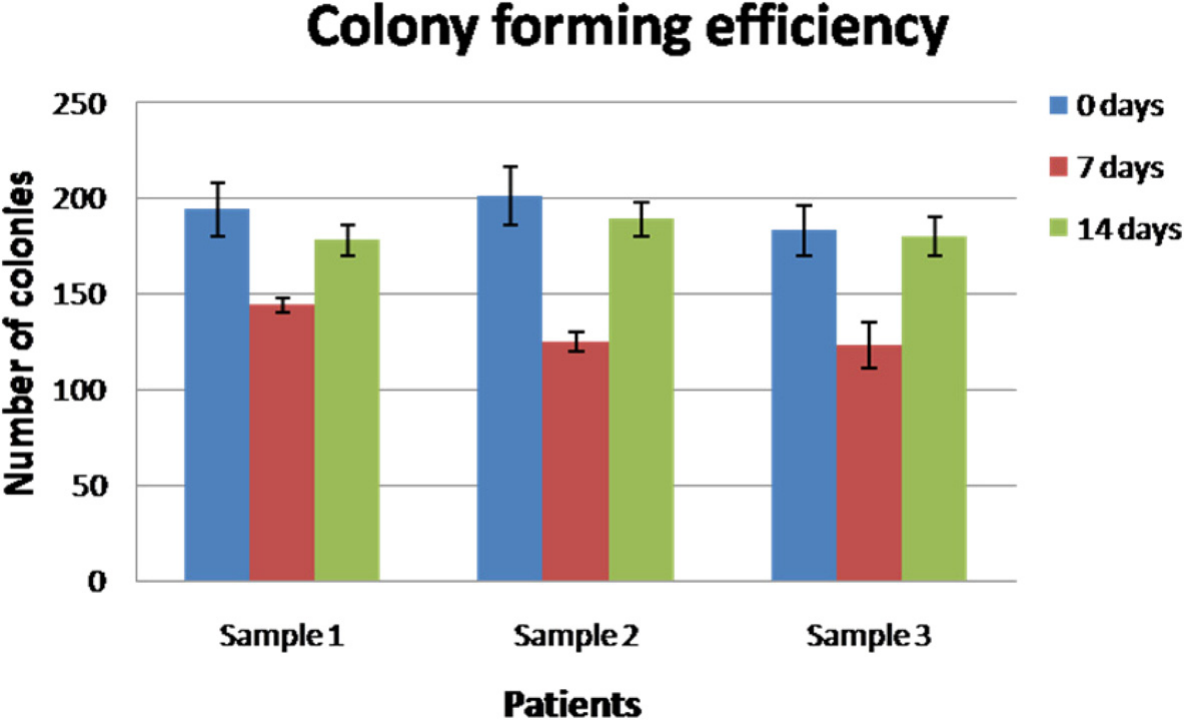
Cell size. Graph representing the percentage of α6β4+/CD71+ cells displaying the same size as the α6β4pos CD71pos oral keratinocyte progenitor cells from pristine sites (each sample identifies different individuals; *n =* 5, *p* <0.05 Student’s *t*-test)

Following biopsies at different time points, the oral keratinocyte progenitor cells were assayed for their cell size. After 0, 7 and 14 days, the percentage of cells displaying the same size as the oral keratinocyte progenitor cells from pristine sites was calculated: Sample 1–100 vs. 68 ± 8.2 vs. 84 ± 4.3; Sample 2–100 vs. 75 ± 5.2 vs. 81 ± 5.1; Sample 3–100 vs. 73 ± 3.1 vs. 79 ± 2.7 for each sample and time point respectively (*n =* 5, *p*<0.05 Student’s *t*-test) (Fig. 4).

#### Immunofluorescence

**Fig. 5:**
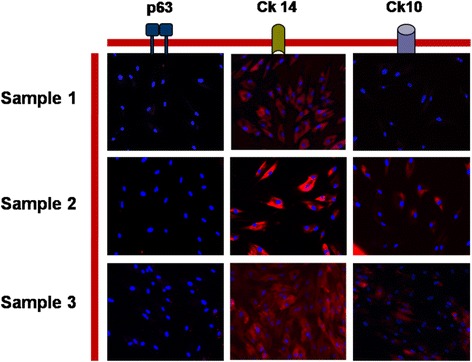
Immunofluorescence. Oral keratinocyte progenitor cells at 14 days expressed different stem- and differentiation markers (nuclei stained with DAPI); original magnification (x40). p63 - a specific oral keratinocyte stem cells marker; CK14 - differentiation marker usually positive for keratinocytes found in the basal layers of the epithelia; CK10 - differentiation marker expressed on cells found in the upper layers of the oral epithelia

After 14 days of *in vitro* culture, actively amplifying cells were characterized by immunostaining using three markers: a keratinocyte stem cell marker - p63; CK14 for basal layer and CK10 for upper layer. In all analyzed samples, our selected keratinocyte subpopulation stained negative for p63 and weakly positive for CK10 and positive for CK14, as shown in Fig. 5.

### Lymphocyte assessment

As shown in Table 1, T-lymphocytes expressed similar viability levels in both the control groups (cells analyzed immediately after isolation) and in stimulated cells. At the same time flow cytometry was used for characterizing lymphocyte cells for specific markers. Results (presented in Table 2) show different distributions of CD8+, CD4+, CD69, CD34 or CD25 T cells after 5 days of lymphocyte exposure to the collagen matrix.

**Table 1:** Lymphocytes viability (in percentages) following Annexin V staining at 14 days

	Sample 1 control	Sample 1 + collagen	Sample 2 control	Sample 2 + collagen	Sample 3 control	Sample 3 + collagen
Viable cells	95.6 ± 1.2	95.7 ± 0.4	90.1 ± 2.1	88.6 ± 2.4	76.5 ± 4.3	77.3 ± 2.2
Necrotic cells	4.0 ± 0.2	3.9 ± 1.1	9.8 ± 1.4	11.2 ± 2.1	23.3 ± 4.3	22.4 ± 4.2

The results show that most analyzed immune cells are viable for each analyzed sample, with very few necrotic cells

**Table 2:** Flow cytometry analysis of lymphocyte cell type: cellular group and the percentage of cells found both in control group and after 5 days seeding on the collagen matrix

	Sample 1 control	Sample 1 + collagen	Sample 2 control	Sample 2 + collagen	Sample 3 control	Sample 3 + collagen
Limphocytes T	18.3 ± 2.1	19.0 ± 3.1	65.6 ± 4.3	66.6 ± 2.1	29.6 ± 5.2	40.3 ± 2.7
Non-T cells	39.5 ± 4.6	12.2 ± 0.2	26.86 ± 2.1	21.4 ± 1.3	27.1 ± 4.7	36.7 ± 6.5
T CD8+	49.1 ± 4.3	57.9 ± 6.5	2.64 ± 0.2	2.88 ± 1.1	12.5 ± 3.7	12.8 ± 2.6
T CD4+	13.2 ± 1.2	18.8 ± 3.3	1.83 ± 0.3	1.21 ± 0.3	12.5 ± 1.6	4.0 ± 1.7
CD38	93.8 ± 4.3	94.8 ± 1.2	8.4 ± 4.5	4.8 ± 0.9	53.1 ± 3.3	48.5 ± 3.4
CD69	72.2 ± 3.4	79.0 ± 2.7	5.4 ± 1.5	1.7 ± 0.1	1.1 ± 0.2	8.9 ± 2.3

## Discussion

Oral keratinocytes and their behaviour on contact with artificial biomaterials used for oral soft tissue regeneration have not been thoroughly studied; however, several experimental studies focused in the past on human keratinocytes cultivated on dermal substitutes. Tinois et al. [42] showed that a construct consisting in a type I + III collagen supporting a type IV collagen layer offers an excellent substrate for keratinocytes, favors their anchorage, and favors the formation of the basement membrane in vitro. Other studies aimed at designing tailored biomaterial surfaces to direct keratinocyte morphology, attachment, and differentiation. These features can be incorporated into dermal equivalents and percutaneous implants to enhance the rate of reepithelialization and tissue regeneration [43]. In a similar experimental approach, the generation of site-appropriate tissue in the treatment of mucogingival defects was attempted by cultivating allogeneic cultured keratinocytes and fibroblasts in a living cellular sheet of bovine collagen, which resulted in the generation of tissue that was more site-appropriate than that transplanted from the palate [44]. To our knowledge, this is the first study on the early healing process after gingival augmentation using collagen soft tissue substitutes, to simultaneously assess the behavior of the cells at the surface and the T-lymphocyte response, with additional confirmatory biopsies.

In the present study, we have analyzed several sub-populations of oral keratinocyte cells, isolated with a previously established protocol by our group [40, 45]. We used two cell surface markers: integrin alfa6beta4 and a proliferation related marker CD71 to isolate three cellular subgroups: integrin alfa6beta4- or the postmitotic differentiation cells, integrin alfa6beta4 + CD71- which represented the oral keratinocyte stem cells population and integrin alfa6beta4 + CD71+ identified as TA cells or progenitor cells. Following placement of a 3D resorbable collagen membrane, at different time points (i.e. before surgery, one week after surgery and two weeks after surgery) we have collected biopsies and isolated and characterized the cellular populations present at the site. Following immunofluorescence staining with different stem- and differentiation markers, we concluded that after 7 days and 14 days the majority of cells that repopulate the matrix were actively proliferating cells or progenitor cells with the phenotype integrin alfa6beta4 + CD71+. The relative lower number of oral keratinocyte stem cells as well as postmitotic differentiation cells, compared to the controls (i.e. pre-operative surgery), can be explained by the fact that progenitor cells are among the first ones that migrate and populate the new healing sites; it must also be noted that our analysis included only a short term assessment of the newly formed mucosa, at 7 and 14 days.

Cellular viability analysis showed that both at 7 and 14 days, most of the cells were viable (over 90 % of the total group) without any statistical differences between the analyzed groups (Fig. 2). This shows that in all cases the interaction between the collagen matrix and the keratinocytes was not cytotoxic and that the matrix does not have negative effects on different keratinocyte sub-populations. The proliferative potential of oral keratinocyte progenitor cells was analyzed after plating the cells in culture for 14 days and analyzing their colony forming efficiency (Fig. 3). Consistent with our previous studies, integrin alfa6beta4 + CD71+ cells were found in an actively proliferating state, especially after 14 days, and formed a statistically comparable number of colonies with the progenitor cells isolated prior to the surgical intervention. In all analyzed samples, TA cells showed less colony forming potential when isolated at 7 days after surgery; we can speculate that these cells need at least one week in order to re-gain their initial colony-forming potential. Our data are consistent with the hypothesis that TA cells are the group responsible with generating the bulk of the keratinocyte pool during the healing process. Another test focused on assaying cellular sizes at both one week and two weeks, and compared the results with the initial pre-operatory assessment (Fig. 4). Our results demonstrate that, especially after two weeks, integrin alfa6beta4 + CD71+ cells showed a similar cell size with the cells analyzed prior to surgery from pristine sites. Thus, in all samples an average of 80% of the cells had the same size with the ones analyzed in the initial biopsy. Immunofluorescence staining demonstrated that most cells represent progenitor keratinocytes, staining weakly positive for CK10, intensely positive for CK14 and negative for p63 (Fig. 5) [46–48]. p63 is a well established keratinocyte stem cell marker, CK14 is usually characteristic to cells found only in the supra-basal layers (such as TA cells) while CK10 is found on keratinocytes residing in the upper layers such as post-mitotic differentiating cells. Taken together, our immunostaining findings suggest that most cells populating the resorbable collagen MATRIX are actively proliferating cells (TA cells) normally found only in the supra-basal layers of the epithelia.

A challenge of the present study was the reduced dimensions of the biopsies and the fragility of the tissues; the separation between the part of the sample destined to histology and the part destined to cell cultures was extremely difficult, even using microsurgical instruments and magnifications.

An ethical concern could be raised regarding the potential influence of the harvesting of the biopsies on the healing process. Human biopsies to evaluate the healing after gingival augmentation procedures using autologous or artificial grafts are repeatedly described in the literature [7, 10, 39]. While one of the mentioned studies describes unproblematic harvesting at 1, 2, 3, 4, 6 and 10 weeks after the surgery from the same surgical site [7], the procedure used in the present study uses biopsies taken with utmost care under microsurgical conditions at 1 and two weeks. As demonstrated before the Commission on Research Ethics, the decision on this procedure was taken based on a long surgical experience of the authors and multiple observations of the post-surgical healing process. On the other hand, biopsies of pristine keratinized gingiva were absolutely necessary forthe study’s target comparisons. The observations show that healing after such particular small-dimension biopsies is uneventful, induces no unnecessary suffering for the patient and leave absolutely no trace.

**Fig. 6:**
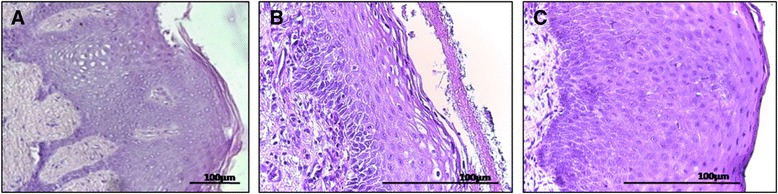
Oral mucosa and collagen matrix. Oral tissue samples before the procedure (**a**)(HE, x10), one week (**b**)(HE, x40) and 2 weeks after the procedure (**c**)(HE, x40). Histological assessment shows that 7 days following surgery, the oral epithelia is still forming, while at 14 days the architecture closely resembles the pristine gingiva (Scale bar - 100μm)

At the same time, histological findings show at baseline normal keratinized gingiva with the well-known mature tissue architecture (Fig. 6a); Fig. 6b displays an incompletely formed keratinized gingiva at 7 days post intervention: the basal third layers of the epithelia are not completely organized while the underlying connective tissue is still regenerating. However, 14 days after the procedure H&E staining demonstrates an oral epithelia that is normally organized with formation of rete ridges and keratinized upper layers, hard to differentiate from the architecture of pristine keratinized gingiva (Fig. 6c).

Another important cell population analyzed for the first time in the present study in relation to its behavior towards the 3D collagen matrix is the T-lymphocytes sub-population, involved in the cell-mediated immunity. Our aim was to assess if the collagen matrix impedes viability, and to characterize this specific cell population for different markers (Table 2). We have analyzed unstimulated T-cells (taken from each patient prior to any surgical intervention) and the same T-cells after culturing them in contact with the collagen matrix for 5 days. The data show that CD8 + T-cells (cytotoxic) increased in number after exposure to the collagen matrix, when compared to matched controls. These results are consistent for all patients included in this study. At the same time, CD4+ T cells decreased, probably as a compensatory mechanism for the citotoxic lymphocytes [49]. CD69 was found to increase in the T-cells adjacent to the collagen matrix; this marker is known to be activated early in the immune response [50]. CD38 data was not consistent for all analyzed samples, but it displayed a statistically non-significant decrease in the immune cells exposed to collagen [50]. At the same time, CD25 was absent in all samples, consistent with other studies that showed that this marker is activated later during the immune response [51]. At the same time, Annexin V staining showed that the percentage of viable lymphocytes was consistent bewteen the control group and the cells exposed to the collagen membrane for each patient, respectively (As shown in Table 1). Data on white blood cells (as presented in Table 2) demonstrates the interpersonal variations in lymphocyte composition (T and non-T lymphocytes, CD8+, CD4+, CD38 and CD69). The differences in T cells composition for patient 1 (Sample 1) may be related to individual characteristics such as age.

One important limitation of the present work is the reduced number of patients and the oral tissue harvesting method, which is currently technique-sensitive and needs further standardization and improvement. Further studies are needed in order to elucidate these issues.

## Conclusion

*In vitro* assessment of different oral keratinocyte subpopulations at 14 days after placing a 3d collagen matrix for augmentation of the keratinized gingiva, with a focus on progenitor cells, supports the claim that maturation of cytologic composition might take more than two weeks, while clinical aspect and histological analysis at the same timepoint demonstrate a mature epithelial architecture. At the same time, the T-lymphocytes response induced by the matrix does not seem to hinder the healing process. However, more long-term studies are needed in order to better analyze the cellular populations involved in regenerating the oral mucosa.
